# Comparing TIMP-1 and Hsp70 in Blood and Saliva as Potential Prognostic Markers in HNSCC

**DOI:** 10.3390/biomedicines10123225

**Published:** 2022-12-12

**Authors:** Jakob Rinecker, Romina Roesch, Sara Krippgans, Markus Nieberler, Leonhard Stark, Stefan Stangl, Bernhard Haller, Kristin Fritsche, Gabriele Multhoff, Andreas Knopf, Christof Winter, Barbara Wollenberg, Markus Wirth

**Affiliations:** 1 Department of Otorhinolaryngology, Head and Neck Surgery, Technical, School of Medicine, University of Munich, 81675 Munich, Germany; 2 Institute of Clinical Chemistry and Pathobiochemistry, School of Medicine, Technical University of Munich, 81675 Munich, Germany; 3 Department of Oral and Maxillofacial Surgery, School of Medicine, Technical University of Munich, 81675 Munich, Germany; 4 Department of Radiation Oncology, School of Medicine, Technical University of Munich, 81675 Munich, Germany; 5 Institute of AI and Informatics in Medicine, School of Medicine, Technical University of Munich, 81675 Munich, Germany; 6 Department of Vascular and Endovascular Surgery, School of Medicine, Technical University of Munich, 81675 Munich, Germany; 7 Department of Otolaryngology Head and Neck Surgery, Albert—Ludwigs-University of Freiburg, 79106 Freiburg im Breisgau, Germany

**Keywords:** TIMP-1, Hsp70, saliva, HNSCC

## Abstract

(1) Background: Currently, there is no clinically used liquid biomarker in head and neck squamous cell carcinoma (HNSCC) patients. One reason could be the limited shedding of tumor material in early disease stages. Molecular diagnostics assessing both blood and especially saliva could potentially improve the accuracy of biomarkers. In this prospective study, two markers, tissue inhibitor of metalloprotease-1 (TIMP-1) and heat shock protein 70 (Hsp70), were analyzed in HNSCC patients. The purpose of the study was to evaluate differences between saliva and serum as sample material. Further, their prognostic and predictive value and usefulness for early detection was assessed. (2) Methods: A total of 73 HNSCC patients were prospectively monitored by collecting blood and saliva before, during, and after therapy, as well as in the follow-up period between 2018 and 2021. In total, 212 serum and 194 saliva samples were collected. A control group consisting of 40 subjects (15 patients with local infections in the head and neck area and 25 without infections) were examined as well. The collected samples were evaluated for the two proteins by using an enzyme-linked immunosorbent assay (ELISA). (3) RESULTS: The TIMP-1 concentration correlated significantly in blood and saliva, whereas the Hsp70 concentration did not. Saliva TIMP-1 was significantly higher in tumor patients compared to the control group (*p =* 0.013). High pretreatment TIMP-1 saliva levels were associated with significantly poorer disease-free survival (DFS) (*p =* 0.02). A high saliva TIMP-1/Hsp70 ratio was significantly associated with poorer DFS (HR: 1.4; 95% CI: 1.04–1.88; *p* = 0.026) and a high TIMP-1 serum concentration was significantly associated with poorer PFS (HR: 1.9; 95% CI: 1.2, 2.8; *p* = 0.003) and poorer overall survival (OS) (HR: 2.9; 95% CI: 1.4, 5.9; *p* = 0.003) in the Cox proportional hazards model. The saliva TIMP-1 to Hsp70 ratio was significantly higher at the time of recurrence (*p =* 0.015). Conclusion: TIMP-1 in serum is a promising prognostic marker for HNSCC. Saliva TIMP-1 and the saliva TIMP-1 to Hsp70 ratio provides additional information on the disease-free survival.

## 1. Introduction

Head and neck squamous cell carcinoma (HNSCC) is the seventh most common cancer entity with around 890,000 new cases and around 450,000 related deaths in 2018 [1,2]. The major risk factors are tobacco and alcohol abuse [2]. Moreover, in oropharyngeal squamous cell carcinomas (OPSCC), tumors that are associated with high-risk human papillomavirus (HPV) infection are increasing [2]. In recent years, progress in treating HNSCC has been rather slow, with stable 5-year overall survival rates of HNSCC patients. Additionally, around 60% of oral cavity and pharyngeal carcinoma are being diagnosed in UICC (Union for International Cancer Control) Stage IV [3]. A better understanding of the individual risk profile could improve diagnostics, treatment, and follow-up algorithms. Liquid biopsies such as circulating tumor cells (CTCs) and circulating tumor DNA (ctDNA) are promising new diagnostic tools [4]. These tools still face limitations hindering clinical application, such as low concentration and sensitivity for CTCs [4]. Moreover, ctDNA is mainly applied for the virus-associated subgroup by detecting HPV-DNA and EBV-DNA or requires genome sequencing to personalize ctDNA assays [4,5]. Recent work focusing on prostate and colorectal cancer showed the additional value of combining conventional biomarkers, which are often cheaper and easier to access, with more specific and complex liquid biopsies [6].

Most commonly, blood is being used as a sample material to measure clinical biomarkers. As saliva can be collected non-invasively, it has been increasingly recognized as a biofluid in recent years [4]. Moreover, saliva offers the advantage of higher biomarker concentration and the possibility to differentiate between a localized or more systemic disease state [7,8]. It is especially interesting for diseases of the head and neck area as saliva plays an important role in protecting the oral epithelium [9]. The actual value of analyzing saliva biomarkers remains unclear due to a lack of studies directly comparing protein markers in serum and saliva in HNSCC. 

In this study, the concentration of two proteins, Hsp70 and TIMP-1, was measured both in serum and saliva. The proteins Hsp70 and TIMP-1 are linked to the tumor microenvironment in HNSCC patients and are secreted by the salivary glands [10,11]. TIMP-1 was found both in acinar and ductal cells [11]. Additionally, TIMP-1 is produced and secreted by gingival fibroblasts [12]. Moreover, mechanisms of blood–saliva transfer of TIMP-1 such as leakage may contribute to a small fragment of the TIMP-1 concentration in saliva [11]. Hsp70 is not secreted by exocytosis in acinar cells but was found in higher concentrations in the striated duct cells of the salivary glands [10]. The main source for extracellular Hsp70 is lysosomal exocytosis, with its regulation associated with CD63 (LAMP3) expression [13].

TIMP-1 influences the tumor microenvironment through different pathways [14]. It is involved in creating a metastatic niche by inducing survival signals [14]. The key aspects are the inhibition of both MMPs (matrix metalloproteinase) and ADAM10 leading to a reorganization of the extracellular matrix and its protease-independent ability to bind CD63 (LAMP3) and ß-Integrin [15]. High TIMP-1 is generally associated with poor cancer outcomes [14]. Additionally, TIMP-1 was found to predict the therapeutic response to several therapeutic modalities such as endocrine therapy in breast cancer and anthracycline-containing chemotherapy in pancreatic ductal carcinoma [16]. In HNSCC, independent studies showed poor overall survival associated with high serum TIMP-1 in oropharyngeal-SCC and high tissue TIMP-1 in laryngeal-SCC [17,18]. To the best of the authors knowledge, saliva TIMP-1 has not been tested in HNSCC so far.

Hsp70 is a heat shock protein that is ubiquitously found and most known for its chaperone activity in folding and remodeling processes. It also acts at the membrane and extracellularly, for example, by stimulating the immune system [19,20]. Depending on its subcellular location, membrane-bound Hsp70 has the ability to either mediate resistance to radiochemotherapy or act as a recognition structure for CD56^+^ NK cells [21]. In serum, it is found during conditions that are linked to stress such as inflammation, infections, and oncological diseases [20]. Additionally, a higher concentration of soluble Hsp70 was measured in the serum of HNSCC patients compared to healthy controls [22]. Hsp70 in saliva has been linked to a host defense mechanism by its immune-activating effect, as part of DAMP (damage-associated molecular patterns), which further activates TLR4 and increases TNFα [10,13]. However, to our knowledge the diagnostic value of Hsp70 has not been tested in saliva of HNSCC patients so far. 

The aim of this study is to compare blood and saliva as sample materials for TIMP-1 and Hsp70 in HNSCC patients during diagnosis, therapy, and follow-up. Further the potential prognostic value of the markers TIMP-1 and Hsp70 both in serum and saliva is analyzed.

## 2. Materials and Methods

**Figure 1:**
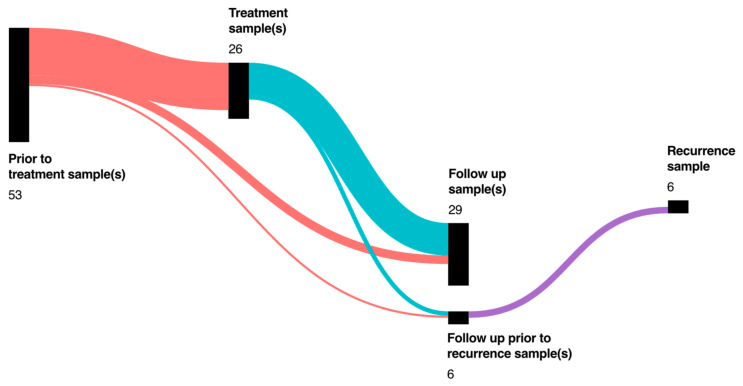
Number of HNSCC patients (total *n* = 73) with one or more sample collections at different timepoints of treatment and follow-up. The size of the bar represents the number of patients at a timepoint. Lines between the bars represent patients with both timepoints in the study timeline.

A single-center study was conducted with 73 HNSCC patients. The patients were recruited at the Klinikum Rechts der Isar, the university hospital of the Technical University of Munich. Generally, only patients over 18 years that gave informed consent were included. The criteria of inclusion for the HNSCC group was defined as squamous cell carcinoma in the oral cavity (including the keratinized part of the lip), as well as oro- (including tonsils), naso- (including paranasal sinus), hypo-, and laryngopharynx. All patients with a secondary malignoma in a different localization or of a different entity were excluded. Sample collection started before the initial therapy (*n* = 53 patients) and during the initial therapy (*n* = 3 Patients), or the follow-up (*n* = 17) (Figure 1). For prognostic analysis of disease-free survival (DFS), progression-free survival (PFS), and overall survival (OS) only prospectively collected samples of the 53 HNSCC patients with a pretreatment sample were included.

If feasible, serum and saliva samples were collected at the same timepoints. The therapeutic interval was defined as the time of treatment plus six months (in the case of radiotherapy, chemotherapy, or immunotherapy) or plus two months (in the case of primary tumor resection). Afterward, the samples were collected during regular follow-up appointments. The median follow-up observation time of the 53 HNSCC-patients with samples before treatment was 19 months with a range from 5 to 40 months. In total, 212 serum and 194 saliva samples were collected, accounting for an average of three serum and saliva samples per patient. The majority, 75% (*n* = 55), of HNSCC patients were males while in the control group male subjects accounted for 62.5% (*n* = 25). The mean age at the time of inclusion was 63.5 years for HNSCC patients and 59 years for the subjects of the control group. A total of 60 tumors were immunohistochemically stained for p16 due to their localization and clinical presentation, resulting in 28 p16-positive and 32 p16-negative tumors. A total of 73.5% of OPSCC were p16^+^. 

A control group consisting of 40 subjects without a history of active malignant disease was also analyzed. The control group consisted of 15 patients with acute or chronic local infections and inflammation in the head and neck region as well as 25 patients without infections. For both control subgroups, a history of any cancer including premalignancies was an exclusion criteria. All the patients were over 18 years old and approved participation in the study by informed consent. The samples were collected at a singular timepoint. The clinical characteristics of HNSCC patients and control subjects are depicted in Table 1. Additionally, the local infections of the control group with infection in the head and neck area are depicted in Supplementary Table S1.

Due to circadian and prandial influence on saliva secretion, the first sample at inclusion was taken in the morning before breakfast. During the follow-up period, the time of collection and the prandial status of the patient were assessed by a questionnaire.

To collect saliva, a slightly modified version of the protocol that was previously published by Wang et al. was used [23]. Therefore, saliva was collected as lavage by advising the patient to keep 10 mL of 0.9% NaCl saline in the mouth for 30 s, gently moving it, before spitting it into a collection tube. Both the serum and saliva were centrifuged at 2000× *g* for 15 min at 15 °C, then the supernatant was stored at −80 °C until further processing.

To collect serum samples, blood was drawn into standardized collection tubes (S-Monovetten serum or serum gel tube, Saarstedt AG & Co., D-51582 Nümbrecht, Germany). After 20–30 min for complete activation of coagulation, the blood samples were centrifuged at 2000× *g* for 15 min at 15 °C, afterwards the supernatant was stored at −80 °C.

The protein concentration was determined using an enzyme-linked immunosorbent assay (ELISA) kit (Human TIMP-1 DuoSet ELISA and Human HSP70/HSPA1A DuoSet ELISA, R&D Systems, Minneapolis, MN USA 55413). The proteins Hsp70 and TIMP-1 were measured in the serum and saliva supernatant. All the measurements were executed in duplicates and performed twice. The concentration was determined through a four-parameter logistic curve fit of a standard concentration dilution series. 

The used Hsp70 ELISA was limited to measure free Hsp70 (fHsp70) and not exosomal Hsp70. Unless stated otherwise, the results refer to fHsp70. For statistical analysis, the Hsp70 concentrations under the detection limit were set to 0.045 ng/mL, which is half of the lowest detectable concentration.

The collected data were statistically analyzed using the open-source software R (https://cran.r-project.org/, accessed on 24 June 2022) and corresponding packages (Supplementary Table S1). For survival analysis, HNSCC patients with pretreatment samples were assessed for overall survival (OS), disease-free survival (DFS), and progression-free survival (PFS). OS was measured from inclusion, reflecting the time of diagnosis of HNSCC, to a patient’s death of any kind. DFS was measured from the end of therapy to disease recurrence or death, excluding patients without remission. PFS was measured from inclusion, reflecting the time of diagnosis until any progression of the disease or death. To compare two independent groups regarding continuously and normally distributed variables, a two sample t-test was used. If the requirement of normal distribution was not met, a Wilcoxon–Mann–Whitney U-test was performed. For comparisons of more than two groups, a Kruskal–Wallis test was used to compare the distributions of relevant variables between the groups.

The correlation of two parameters was assessed using the Spearman correlation coefficient and interpreted as published in Schober et al. (2018) [24]. Cut-off values were either used from the literature or established by using the median of the variable. Survival curves were visualized using Kaplan–Meier curves and tested for significant differences between groups by using the logrank test. To estimate associations between the potential prognostic variables and time-to-event endpoints (OS, PFS, DFS), Cox proportional-hazards regression models were fitted to the data (with 10^2^ ng as unit for TIMP-1 and Hsp70 and 10^1^ units for TIMP/Hsp70). In general, *p*-values that were smaller than 0.05 were considered statistically significant.

## 3. Results

### 3.1. TIMP-1 and Hsp70 in Serum and Saliva

Hsp70 in saliva (mean at inclusion: 15.9 ng/mL) was on average 55 times higher than in serum (mean at inclusion: 0.29 ng/mL). The opposite was the case for TIMP-1, where the saliva concentration (mean at inclusion: 27.1 ng/mL) was measured to be 43% of the serum concentration (mean at inclusion: 63.3 ng/mL). In the 40 subjects and the 53 pretreatment samples of HNSCC patients, the serum and saliva concentrations of TIMP-1 revealed a weak positive correlation (rho = 0.3, *p* = 0.004), while no significant correlation was seen for Hsp70 in the serum and saliva (Table 2). In a subgroup analysis, the positive correlation between TIMP-1 in serum and saliva was only detected in pretreatment HNSCC subjects (rho = 0.34, *p* = 0.015), but not in the control group (rho = 0.24, *p* = 0.13). In the follow-up, ingestion of food and beverages was documented and the association of fluid intake and food intake with the markers was analyzed. The Hsp70 saliva concentration revealed a significant moderate positive correlation (rho = 0.42, *p* = 0.04) with the time since the subjects last consumed a fluid. Overall, no further association between fluid or food intake was measured for any of the two markers.

Hsp70 and TIMP-1 did not correlate in serum (rho = −0.12, *p* = 0.27) but correlated moderately in saliva (rho = 0.47, *p* < 0.001). In a subgroup analysis, this was true for the control patients (*n* = 40, rho = 0.51, *p* < 0.001) and patients with UICC Stage ≤ II (*n* = 25, rho = 0.52, *p* < 0.001), but there was no significant correlation for UICC Stage > II (*n* = 25, rho = 0.38, *p* = 0.07).

To compare the overall properties of the markers, the relative standard deviation (SD) was calculated for each patient with at least two samples of each sample material (*n* = 29). The SD of TIMP-1 was lowest in the serum with 21.9% compared to 56.5% in saliva. For Hsp70, the relative SD was 88.5% in serum and 81.4% in saliva. The detailed relative SD can be found in Supplementary Table S1.

### 3.2. Comparing Subjects with HNSCC and Control Subjects with and without Infection

**Figure 2:**
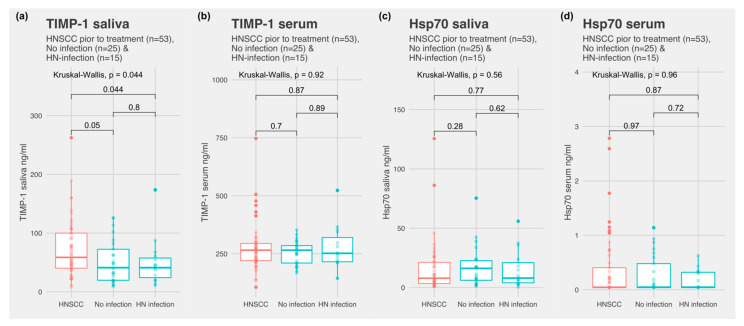
(**a**) TIMP-1 saliva concentration in subjects with HNSCC, control subjects without infection, and control subjects with head and neck infection; (**b**) TIMP-1 serum concentration in subjects with HNSCC, control subjects without infection, and control subjects with head and neck infection; (**c**) Hsp70 saliva concentration in subjects with HNSCC, control subjects without infection, and control subjects with head and neck infection; and (**d**) Hsp70 serum concentration in subjects with HNSCC, control subjects without infection, and control subjects with head and neck infection.

**Figure 3:**
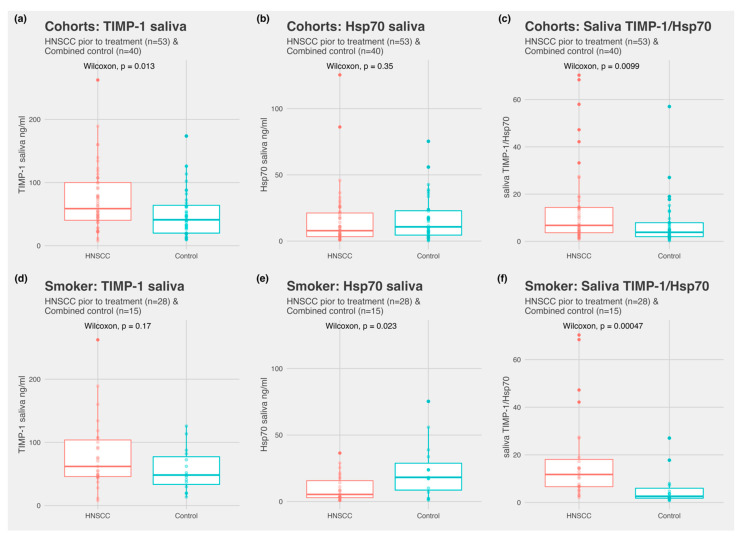
Comparing pretreatment HNSCC (*n* = 53) and combined control subjects (*n* = 40) for TIMP-1 and Hsp70 both in serum and saliva. (**a**) TIMP-1 saliva concentration in HNSCC and combined control subjects; (**b**) Hsp70 saliva concentration in HNSCC and combined control subjects; (**c**) TIMP-1/Hsp70 saliva ratio comparing HNSCC and combined control subjects; (**d**) saliva TIMP-1 in smokers with HNSCC and combined smoking control subjects; (**e**) saliva Hsp70 in smokers with HNSCC and combined smoking control subjects; and (**f**) TIMP-1/Hsp70 ratio in smokers with HNSCC and combined smoking control subjects.

Patient characteristics are depicted in Table 1, Table 2 and Table 3. TIMP-1 was detected in all the samples, both in serum and saliva. Hsp70 was detected in all the saliva samples. In serum, Hsp70 was detectable in 23 of 53 HNSCC subjects, 7 of 15 subjects with infection, and 12 of 25 subjects without infection. There was no significant difference between control subjects with an infection and control subjects without infection for all the markers in both fluids (Figure 2). Subjects with HNSCC revealed significantly higher TIMP-1 values in saliva compared to the control subjects without infections (*p =* 0.05) and the combined control subjects (*p* = 0.013) (Figure 3a). Hsp70 in saliva did not differ significantly between the two groups. The ratio of TIMP-1/Hsp70 was significantly higher in HNSCC patients compared to the control subjects without infections (*p =* 0.014) and combined control group (*p* = 0.0099). In serum, TIMP-1 and Hsp70 revealed no overall difference between the groups.

Receiver operator curves were performed for all the markers (Supplementary Figure S1), with further calculation of the AUC (area under the curve) to compare the overall performance of distinguishing HNSCC and the combined control group. The AUC measured 0.65 for saliva TIMP-1, 0.55 for saliva Hsp70, and 0.66 for the saliva ratio TIMP-1/Hsp70. The AUC of serum TIMP-1 and serum Hsp70 was calculated to be under 0.55. In a subgroup analysis compared to combined control group for the saliva ratio TIMP-1/Hsp70, the AUC was 0.8 for UICC Stage IV patients and 0.7 for HPV-negative tumors. A smoker-only subgroup showed an AUC of 0.8 for smokers with HNSCC compared to smokers of the combined control group.

### 3.3. Marker Association with Clinical and Pathological Parameters

The association between the relevant clinical parameters and initial Hsp70 and TIMP-1 measurements for 53 HNSCC patients with pre-treatment samples are depicted in Table 3 and Table 4. Serum TIMP-1 was significantly higher in patients with UICC Stage >II. A weak negative correlation was observed between the Hsp70 saliva concentration and both smoking (rho = −0.3, *p* = 0.04) and the regular use of alcohol (regularly, ex, no rho = −0.33, *p* = 0.04). In the control group, Hsp70 in saliva showed a moderate positive correlation with smoking (current, ex, no; rho = 0.4, *p* = 0.03), whereas serum Hsp70 was weakly positively correlated with the use of alcohol (current, ex, no; rho = 0.36, *p* = 0.04). In a subgroup analysis, a significantly lower Hsp70 saliva concentration (*p* = 0.023) was found in smokers with HNSCC compared to smokers in the combined control group. This association was also detected in the smoker subgroup for the TIMP-1/Hsp70 ratio (*p* = 0.0005) but not in the non-smoker subgroup (*p* = 0.5; Supplementary Figure S1).

### 3.4. Prognostic Value of the Protein Markers

The Cox proportional hazards ratio (CoxPH) model revealed serum TIMP-1 to be significantly associated with both progression-free survival (TIMP-1 serum/PFS; HR: 1.9; 95% CI: 1.2, 2.8; *p* = 0.003) and overall survival (TIMP-1 serum/OS, HR: 2.9; 95% CI: 1.4, 5.9; *p* = 0.003). Further, the CoxPH model of the ratio between TIMP-1 and Hsp70 in saliva (TIMP-1/Hsp70) was significantly associated with disease-free survival (DFS) (TIMP-1/Hsp70 saliva ratio/DFS, HR: 1.4; 95% CI: 1.04–1.88; *p* = 0.026) but DFS was not significantly associated with singular TIMP-1 or Hsp70 in saliva.

The median was used to define high- and low-value groups for saliva markers (cut-off: 62.8 ng for TIMP-1 and cut-off 7.9 ng for Hsp70 saliva) since neither TIMP-1 or Hsp70 have been tested in saliva so far. To determine the prognostic value of markers, the pre-treatment values of HNSCC patients that were treated with curative intent (*n* = 48) were analyzed. Patients with high saliva TIMP-1 had a significantly worse DFS (*p =* 0.02; Figure 2a) but not overall survival (OS) (*p =* 0.1; Supplementary Figure S1). TIMP-1 serum cut-off (196 ng/mL) established by Carpen et al. [17] was not significantly associated with DFS (*p* = 0.1) or OS (*p* = 0.3) in our cohort (Supplementary Figure S1).

### 3.5. Follow-Up

**Figure 4:**
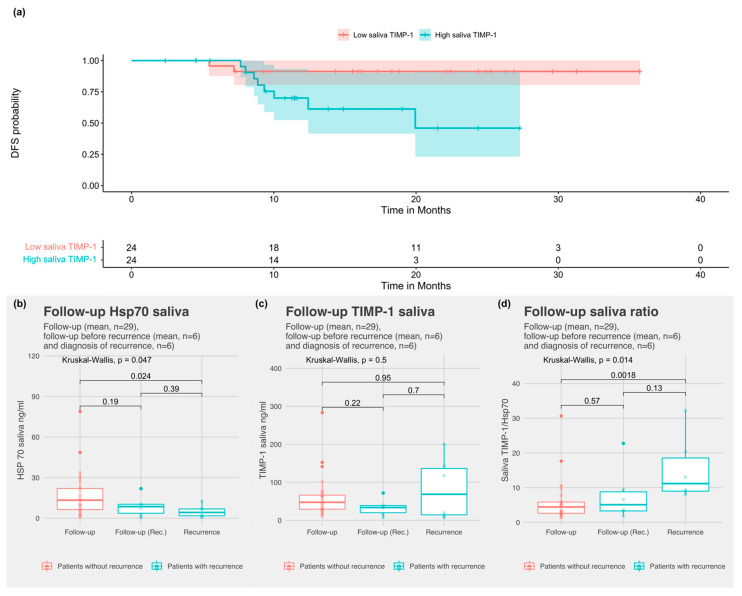
(**a**) Disease-free survival probability for high and low saliva TIMP-1 by the median cut-off of 62.8 ng; (**b**) comparison of saliva Hsp70 during follow-up (mean per subject) for HNSCC-patients without recurrence, before recurrence, and levels at the time of recurrence; (**c**) comparison of saliva TIMP-1 during follow-up (mean per subject) for HNSCC-patients without recurrence, before recurrence, and levels at the time of recurrence; and (**d**) comparison of saliva TIMP-1/Hsp70 ratio during follow-up (mean per subject) for HNSCC patients without recurrence, before recurrence, and levels at the time of recurrence).

Hsp70 saliva levels in patients with recurrent cancer were significantly lower than the average follow-up levels of patients without recurrence (*p =* 0.047) (Figure 4b). The TIMP-1 saliva concentration was not increased in patients with recurrence (*p* = 0.5). The TIMP-1/Hsp70 ratio in saliva was significantly higher at recurrence compared to the follow-up (*p* = 0.014) (Figure 4d). The mean TIMP-1/Hsp70 saliva ratio was higher six months prior to recurrence (including the time of diagnosis of disease recurrence) than the mean follow-up (*p* = 0.051, Supplementary Figure S1). 

## 4. Discussion

In this study, the use of serum and saliva as a potential source for the protein markers TIMP-1 and fHsp70 was analyzed, hereby assessing different aspects such as the difference between tumor patients and healthy subjects as well as the prognostic value of the markers. Saliva TIMP-1 was significantly higher in HNSCC patients compared with control subjects. Both markers, TIMP-1 and fHsp70, were not significantly impacted by oral or head and neck infections. Serum and saliva TIMP-1, as well as the saliva TIMP-1/fHsp70 ratio were prognostic markers for the patient’s outcome.

Serum TIMP-1 was significantly associated with poorer OS and poorer PFS in our cohort in the Cox proportional hazards ratio model. This is in accordance with previous studies in HPV-negative OPSCC and HNSCC [17,25]. TIMP-1 has been attributed to play a role in creating a metastatic niche by interacting with the tetraspanin CD63 (LAMP3) surface receptor [15]. This leads to survival signals by the activation of focal adhesion kinase (FAK), phosphatidylinositol 3-kinase (PI3K), and extracellular signal-regulated kinase (ERK) [14,15]. Further, this TIMP-1-CD63 (LAMP3) signaling axis has been identified as playing a role in tumor metabolism of breast cancer cells by downstream upregulating aerobic glycolysis through carbonic anhydrase IX (CAIX) and thereby extending cell survival [26]. Creating this metastatic niche could be a factor leading to the observed overall decrease in PFS and OS in patients with high serum TIMP-1 concentration. However, the previously published cut-offs (196 ng/mL for HPV-negative OPSCC, 510 ng/mL for HNSCC) were not significantly associated with survival in our cohort [17,25]. The reasons for the different overall medians in tumor-sample measurements could be the use of different antibodies and kits, although all the measurements were assessed by ELISA [17,25]. 

TIMP-1 has not been examined in the saliva of HNSCC patients so far. Therefore, we used the median to differentiate between high and low expressing groups. High saliva TIMP-1 was significantly associated with poorer DFS but not with OS. Additionally, TIMP-1 was significantly increased in saliva but not in serum of HNSCC patients, when compared to the control group. The higher TIMP-1 saliva concentration was presumably detected due to the shedding of the tumor or the tumor microenvironment with direct contact to the oral and pharyngeal cavity. TIMP-1 has been mainly detected in tumor cells, vascular endothelium, and carcinoma-associated fibroblasts in HNSCC, thereby acting at the interface between the tumor microenvironment and the tumor’s systemic connection [27,28]. TIMP-1’s occurrence at this interface could explain why TIMP-1 is a strong prognostic marker in serum and shows significant higher levels in saliva of HNSCC patients. Furthermore, saliva TIMP-1 was weakly correlated with serum values and the correlation increased in the HNSCCC subgroup. In healthy subjects, saliva from the parotid gland is the main source of TIMP-1 secretion as TIMP-1 glycosylation profiles were found to differ greatly in serum and saliva [29]. Therefore, the increase in correlation between saliva and serum in the HNSCC subgroup is potentially a result of the upregulation of TIMP-1 expression in HNSCC, as well as a potential TIMP-1 secretion from cancer cells, as suggested by Caprén et al. [16,25,28].

TIMP-1 in saliva has previously been investigated as a biomarker for periodontal disease, and thereby a significantly lower TIMP-1 saliva concentration was detected [30]. Consequently, a history of periodontal disease was queried in our questionnaire. However, we did not measure a difference for either TIMP-1 and fHsp70, between subjects with previous periodontal disease and those with no history of periodontal disease. However, the self-reported periodontal disease could also be biased since patients were not examined by a dentist as part of the trial. 

The saliva fHsp70 concentration was independent of its serum concentration while being on average 55 times higher in saliva. The comparison between serum and saliva was limited due to serum Hsp70 being under the detection limit in 56.6% of HNSCC patients and 52.5% of the controls. The high Hsp70 saliva concentration compared to serum supports a previously discussed passive or small active transport for Hsp70 into saliva [10]. In the case of an active transport, lysosomal exocytosis or exosome-dependent trafficking could be potential modes of transport into saliva, influenced by the stimulation of saliva-secretion [13,31,32]. CD63/LAMP3 expression in salivary gland cells has been associated with caspase-dependent lysosomal exocytosis of Hsp70 [33]. However it has to be taken into account that the ELISA that was used for this study only detects free Hsp70 but not exosomal Hsp70. Hsp70 acts as a mucosal and periodontal defense [34,35] by being an endogenous natural ligand of TLR4, which plays a key role in inflammation [13,36,37]. TLR4 activation has been described to mediate anti-EGFR therapy resistance in head and neck cancer [36]. However fHsp70 was not associated with poorer outcome in this study.

The saliva fHsp70 concentration negatively correlated with the risk factors alcohol and smoking in HNSCC patients. Conversely, in the control group, saliva fHsp70 was positively correlated with smoking. The positive correlation is in line with Hsp70 being part of the damage-associated molecular patterns (DAMPs), that are induced by cigarette smoke [38,39]. Smokers with HNSCC and without cancer show a greatly different oral microbiome, as smokers with HNSCC present significant lower microbiome richness and higher interindividual microbiome heterogeneity [40]. Consequently, the inverse effect on Hsp70 in smoking HNSCC patients and it’s possible connection with the oral microbiome and oral immune status, respectively, could be a confounding explanatory factor. 

A further influence on fHsp70 concentration in saliva was observed, as it moderately positively correlated to time of last fluid intake. Fluid intake stimulates saliva secretion, which leads to a decrease in Hsp70 saliva concentration [31]. This was only visible during the follow-up period as fluid intake was not prohibited, unlike at the pretreatment and inclusion samples. 

In our cohort, the saliva TIMP-1/fHsp70 ratio was significantly higher for HNSCC patients compared with control patients and higher in samples of patients with recurrence compared to the mean follow-up. Moreover, high saliva TIMP-1/fHsp70 ratio was associated with poorer DFS. We used the saliva TIMP-1 in a ratio with saliva fHsp70 to ameliorate the sensitivity. We explored saliva fHsp70 as a denominator, because of our finding of lower mean fHsp70 concentration in HNSCC patients. Additionally stimulation of saliva secretion is described to decrease Hsp70 and TIMP-1 concentration [11,31]. A ratio potentially reduces this effects. In addition, previous work indicates that CD63/LAMP3 is a cell surface binding partner for TIMP-1 and acts as part of the Hsp70 release mechanism in salivary gland tissue [13,15]. This suggests a potential interaction between TIMP-1 and Hsp70 through CD63 (LAMP3). Also, CD63 (LAMP3) by itself was found to be a prognostic marker in LSCC tissue [41]. 

In both saliva TIMP-1 and the saliva TIMP-1/fHsp70 ratio, we observed particularly high levels at time of recurrence, especially in samples of patients with local recurrence. These data are promising but due to the limited number of observations (recurrence *n* = 6, local recurrence *n* = 4) need further investigation. An important factor is the difference between local and metastatic recurrence and their effect on the marker concentration, which would be interesting to focus on in future studies.

There are several limitations in our study such as the heterogeneous therapy regimes, different follow-up cycles, and missing samples. This occurred in part due to follow-up outside our study center and the COVID pandemic. Some follow-up samples that were not collected prospectively as pretreatment samples were not available for all HNSCC subjects. Especially, the follow-up samples that were taken at different timepoints is an important bias. When analyzing intervals without absolute events such as diagnosis, recurrence, or death, we used the mean values of the time interval. This could only partially minimize the effect of the bias due to different numbers in observations and length of the time intervals.

In conclusion, our data support that serum TIMP-1 is a promising prognostic marker for HNSCC. Furthermore, saliva TIMP-1 and the saliva TIMP-1 to fHsp70 ratio provide additional information on disease-free survival. Still, the main obstacle for protein biomarkers is specificity, which could be addressed through a better understanding of their molecular origins and secretion pathways. In a future study, an analysis of protein glycosylation profiles could be performed. It has been reported that TIMP-1 glycosylation profiles differ greatly between plasma and saliva [29]. Further glycosylation changes in HNSCC have been described for multiple proteins with important effects on cancer stem cells, the epithelial-mesenchymal transition (EMT), and tumor-related immunity escape and autophagy [42]. This information could be used for a more precise quantification of tumor-derived proteins in saliva.

## Figures and Tables

**Table 1:** (**a**) Clinical characteristics of HNSCC patients; (**b**) TNM-staging (according to UICC, 8th edition) of HNSCC patients; and (**c**) Clinical characteristics of control subjects.

(a) HNSCC Patients Characteristics				(b) TNM Staging				(c) Control Subjects Characteristics			
		sample before therapy				sample before therapy				Head/Neck infection	
**Characteristic**	**Overall,**	**no,**	**yes,**	**Characteristic**	**Overall,**	**no,**	**yes,**	**Characteristic**	**Overall,**	**no,**	**yes,**
	***n*** **= 73 ^1^**	***n*** **= 20 ^1^**	***n*** **= 53 ^1^**		***n* = 73 ^1^**	***n* = 20 ^1^**	***n* = 53 ^1^**		***n* = 40 ^1^**	***n* = 25 ^1^**	***n* = 15 ^1^**
**Age at inclusion**	63 (56, 72)	64 (58, 70)	63 (55, 72)	**Staging**				**Age at inclusion**	60 (54, 67)	61 (55, 67)	59 (52, 66)
**Gender**				I	14 (19%)	6 (30%)	8 (15%)	**Gender**			
Female	18 (25%)	5 (25%)	13 (25%)	II	21 (29%)	4 (20%)	17 (32%)	Female	15 (38%)	7 (28%)	8 (53%)
Male	55 (75%)	15 (75%)	40 (75%)	III	18 (25%)	3 (15%)	15 (28%)	Male	25 (62%)	18 (72%)	7 (47%)
**Localization**				IV	19 (26%)	7 (35%)	12 (23%)	**Smoking**			
CUP	4 (5.5%)	0 (0%)	4 (7.5%)	Tis	1 (1.4%)	0 (0%)	1 (1.9%)	Non-smoker	11 (28%)	5 (20%)	6 (40%)
Hypopharynx	6 (8.2%)	1 (5.0%)	5 (9.4%)	**Tumor**				Ex-smoker	5 (12%)	5 (20%)	0 (0%)
Larynx	8 (11%)	5 (25%)	3 (5.7%)	T0	5 (6.8%)	0 (0%)	5 (9.4%)	Smoker	15 (38%)	11 (44%)	4 (27%)
Nasopharynx	2 (2.7%)	1 (5.0%)	1 (1.9%)	T1	12 (16%)	3 (15%)	9 (17%)	Unknown	9 (22%)	4 (16%)	5 (33%)
Oral Cavity	19 (26%)	5 (25%)	14 (26%)	T2	21 (29%)	8 (40%)	13 (25%)	**Alcohol**			
Oropharynx	34 (47%)	8 (40%)	26 (49%)	T3	19 (26%)	3 (15%)	16 (30%)	Not regularly	19 (48%)	11 (44%)	8 (53%)
**p16 IHC**				T4	16 (22%)	6 (30%)	10 (19%)	Regularly	12 (30%)	10 (40%)	2 (13%)
No p16 IHC	13 (18%)	2 (10%)	11 (21%)	**Nodus**				Unknown	9 (22%)	4 (16%)	5 (33%)
p16-	32 (44%)	9 (45%)	23 (43%)	N0	25 (34%)	10 (50%)	15 (28%)	^1^ Median (IQR); *n* (%)			
p16+	28 (38%)	9 (45%)	19 (36%)	N1	21 (29%)	5 (25%)	16 (30%)				
**Smoking**				N2	24 (33%)	4 (20%)	20 (38%)				
Non-smoker	19 (26%)	3 (15%)	16 (30%)	N3	2 (2.7%)	0 (0%)	2 (3.8%)				
Ex-smoker	7 (9.6%)	3 (15%)	4 (7.5%)	Nx	1 (1.4%)	1 (5.0%)	0 (0%)				
Smoker	42 (58%)	14 (70%)	28 (53%)	**Metastasis**							
Unknown	5 (6.8%)	0 (0%)	5 (9.4%)	M0	65 (89%)	14 (70%)	51 (96%)				
**Alcohol**				Mx	1 (1.4%)	0 (0%)	1 (1.9%)				
Not regularly	21 (29%)	7 (35%)	14 (26%)	M1	7 (9.6%)	6 (30%)	1 (1.9%)				
Ex-regularly	1 (1.4%)	0 (0%)	1 (1.9%)	^1^*n* (%)							
Regularly	35 (48%)	8 (40%)	27 (51%)								
Unknown	16 (22%)	5 (25%)	11 (21%)								
^1^ Median (IQR); *n* (%)											

**Table 2:** Spearman correlation (rho) of the markers in saliva and serum (samples of pretreatment HNSCC patients (*n* = 53) and control subjects (*n* = 40).

Marker	Hsp70 Serum	Hsp70 Saliva	TIMP-1 Serum
Hsp70 serum			
Hsp70 saliva	0.01		
TIMP-1 serum	−0.12	0.27 ^1^	
TIMP-1 saliva	−0.03	0.47 ^1^	0.30 ^1^

^1^ indicates *p* < 0.05.

**Table 3:** Protein concentration and staging parameters of HNSCC patient pretreatment samples (*n* = 53).

Staging Parameters						
	Staging			Tumor (T)		
** Concentration (ng/mL) **	** ≤II, *n* = 26 ^1^**	** >II, *n* = 27 ^1^**	***p*-value ^2^**	** ≤II, *n* = 27 ^1^**	** >II, *n* = 26 ^1^**	***p*-value ^2^**
TIMP-1 serum	237 (213, 274)	274 (237, 321)	0.047	263 (212, 282)	270 (233, 314)	0.2
TIMP-1 saliva	55 (37, 78)	68 (45, 107)	0.3	55 (39, 84)	76 (44, 107)	0.6
Hsp70 serum	0.05 (0.05, 0.31)	0.05 (0.05, 0.45)	0.9	0.05 (0.05, 0.41)	0.05 (0.05, 0.36)	0.6
Hsp70 saliva	8 (3, 18)	8 (3, 22)	0.8	9 (3, 22)	7 (3, 21)	>0.9
	** Nodus (N) **			** Metastasis (M) **		
** Concentration (ng/mL) **	** =0, *n* = 15 ^1^**	** ≥I, *n* = 38 ^1^**	***p*-value ^2^**	** 0, *n* = 51 ^1^**	** X or I, *n* = 2 ^1^**	***p*-value ^3^**
TIMP-1 serum	259 (222, 285)	266 (221, 295)	0.8	267 (220, 295)	239 (232, 246)	0.5
TIMP-1 saliva	54 (42, 85)	67 (39, 104)	0.6	62 (41, 100)	23 (23, 23)	0.3
Hsp70 serum	0.13 (0.05, 0.23)	0.05 (0.05, 0.60)	0.8	0.05 (0.05, 0.37)	0.43 (0.33, 0.53)	0.2
Hsp70 saliva	11 (5, 24)	7 (3, 19)	0.2	8 (3, 22)	5 (5, 5)	0.8

^1^ Median (IQR); ^2^ Wilcoxon rank sum exact test; Wilcoxon rank sum test; ^3^ Wilcoxon rank sum test; Wilcoxon rank sum exact test.

**Table 4:** Protein concentration and pathological and clinical parameters of HNSCC patient pretreatment samples (n = 53).

**Pathological and Cliniacal Parameters**				
	**p16 IHC**			
** Concentration (ng/mL) **	** no p16 IHC, *n* = 11 ^1^**	** p16-, *n* = 23^1^**	** p16+, *n* = 19 ^1^**	***p*-value ^2^**
TIMP-1 serum	274 (264, 285)	267 (219, 367)	236 (218, 277)	0.2
TIMP-1 saliva	90 (46, 113)	51 (44, 81)	67 (37, 89)	0.6
Hsp70 serum	0.05 (0.05, 0.13)	0.13 (0.05, 0.67)	0.05 (0.05, 0.48)	0.4
Hsp70 saliva	8 (3, 26)	5 (2, 15)	11 (5, 24)	0.2
	**Smoking**			
** Concentration (ng/mL) **	** non-smoker, *n* = 16 ^1^**	** ex-smoker, *n* = 4 ^1^**	** smoker, *n* = 28 ^1^**	***p*-value ^2^**
TIMP-1 serum	236 (223, 288)	232 (215, 246)	271 (220, 305)	0.3
TIMP-1 saliva	67 (32, 103)	46 (37, 58)	62 (46, 104)	0.5
Hsp70 serum	0.05 (0.05, 0.19)	0.14 (0.05, 0.87)	0.05 (0.05,0.43)	0.9
Hsp70 saliva	17 (6, 26)	5 (5, 7)	5 (3, 16)	0.11
	**Alcohol**			
** Concentration (ng/mL) **	** not regularly, *n* = 14 ^1^**	** ex-regularly, *n* = 1 ^1^**	** regularly, n = 27 ^1^**	***p*-value ^2^**
TIMP-1 serum	250 (214, 273)	267 (267, 267)	274 (224, 331)	0.3
TIMP-1 saliva	52 (40, 106)	62 (62, 62)	76 (48, 100)	0.8
Hsp70 serum	0.13 (0.05, 0.30)	1.15 (1.15, 1.15)	0.05 (0.05, 0.45)	0.2
Hsp70 saliva	16 (5, 26)	9 (9, 9)	6 (3, 11)	0.12

^1^ Median (IQR); ^2^ Kruskal-Wallis rank sum test.

## Data Availability

The data that are presented in this study are available in the Supplementary Materials.

## Supplementary Materials

The following supporting information can be downloaded at: https://www.mdpi.com/article/10.3390/biomedicines10123225/s1, Figure S1: (a) Receiver operator characteristic curve for saliva TIMP-1 classifying HNSCC patients (*n* = 53) and control subjects (*n* = 40), (b) Receiver operator characteristic curve for saliva Hsp70 classifying HNSCC patients (*n* = 53) and control subjects (*n* = 40), (c) Receiver operator characteristic curve for serum TIMP-1 classifying HNSCC patients (*n* = 53) and control subjects (*n* = 40), (d) Receiver operator characteristic curve for serum Hsp70 classifying HNSCC patients (*n* = 53) and control subjects (*n* = 40), (e) Receiver operator characteristic curve for TIMP-1/Hsp70 saliva ratio classifying HNSCC patients (*n* = 53) and control subjects (*n* = 40), (f) Receiver operator characteristic curve for TIMP-1/Hsp70 saliva ratio classifying HNSCC patients in smoker subgroup with smokers with HNSCC (*n* = 28) & smokers control (*n* = 15) (g) Receiver operator characteristic curve for TIMP-1/Hsp70 saliva ratio classifying HNSCC patients in a subgroup with Nodus state N3 (*n* = 6) & control (*n* = 40), (h) Receiver operator characteristic curve for TIMP-1/Hsp70 saliva ratio classifying HNSCC patients in a subgroup with UICC Stage IV (*n* = 6) & control (*n* = 40), (i) Receiver operator characteristic curve for TIMP-1/Hsp70 saliva ratio classifying HNSCC patients in a subgroup with HPV negative (*n* = 34) & control (*n* = 40), (j) Saliva ratio TIMP-1 in non-smoking subjects, comparing HNSCC and the combined control group, (k) Saliva ratio Hsp70 in non-smoking subjects, comparing HNSCC and the combined control group, (l) Saliva ratio TIMP-1/Hsp70 in non-smoking subjects, comparing HNSCC and the combined control group, (m) PFS probability for TIMP-1 concentration above (high) and below (low) cut off value 196ng/ml, (n) OS probability for TIMP-1 concentration above (high) and below (low) cut off value 196ng/ml, (o) Saliva TIMP-1 concentration mean during follow up, follow up before local recurrence and concentration at diagnosis of local recurrence, (p) Saliva TIMP-1/Hsp70 ratio mean during follow up, follow up before local recurrence and at diagnosis of local recurrence, (q) Saliva TIMP-1/Hsp70 ratio mean during follow up, follow up before metastatic recurrence and concentration at diagnosis of metastatic recurrence, (r) Saliva TIMP-1/Hsp70 ratio mean in follow up, follow up before metastatic recurrence and concentration at diagnosis of metastatic recurrence, (s) TIMP-1/Hsp70 saliva ratio mean during follow up and mean six months prior to recurrence. Table S1: (a) Table of relative standard deviation of marker concentration in HNSCC patients, control subjects without infection with at least 2 samples per material (b) Table of the subgroup with head and neck infections (*n* = 15) and their diagnosis of infection at time of sample collection. Localization specified for the different types of infections. (c) R-packages used for statistical analysis and visualization.
