# Supplementary material for: Comparing TIMP-1 and Hsp70 in Blood and Saliva as Potential Prognostic Markers in HNSCC
Source: Biomedicines. 2022 Dec 12;10(12):3225. doi: 10.3390/biomedicines10123225 (PMC9775946; doi:10.3390/biomedicines10123225)
Supplement: Supplementary file 1 [file biomedicines-10-03225-s001.zip › biomedicines-2035943-supplementary.pdf]

information

MDPI

Supplementary Table S1. (a) Table of relative standard deviation of marker concentration in HNSCC patients, control subjects without infection with at least 2 samples per material (b) Table of the subgroup with head and neck infections (*n*=15) and their diagnosis of infection at time of sample collection. Localization specified for the different types of infections. (c) R-packages used for statistical analysis and visualization.

Variable	TIMP-1 serum	TIMP-1 saliva	Hsp70 serum	Hsp70 saliva
**Per Patient (n=29, ≥2 samples)**	21.9%	56.5%	88.5%	81.4%
**HNSCC patients (n=52)**	37.5%	68.5%	182.7%	142.2%
**Subjects without infection (n=25)**	19.8%	70.1%	123.5%	97.8%

(a)

Diagnosis of subgroups with infections	localization	number = n
**abscess**	peritonsilliar, tongue, jaw (*n*=2)	4
**purulent sialadenitis**	salivary gland	1
**erysipelas**	temporal scalp	1
**laryngitis**	larynx	2
**osteonecrosis**	lower jaw (*n*=2)	2
**cellulitis**	orbital, base of the mouth	2
**sinusitis**	paranasal sinus	1
**tonsillitis**	tonsills	2

(b)

R-packages
datasets, car, pacman
tidyverse, dplyr
psych, survival, survminer
ggplot2, ggthemes, ggrepel, ggsignif, ggpubr
pROC, gridExtra, finalfit
gtsummary, devtools

(c)

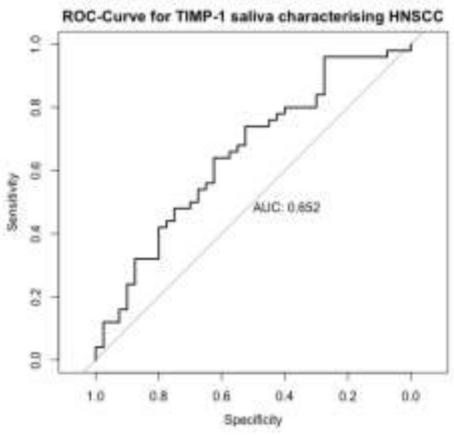
ROC-Curve for TIMP-1 saliva characterising HNSCC
AUC: 0.652
Sensitivity
Specificity

(a)

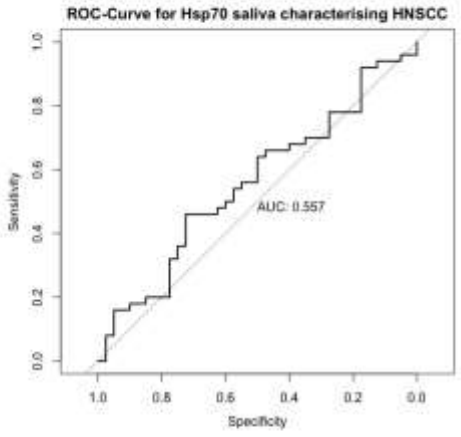
ROC-Curve for Hsp70 saliva characterising HNSCC
AUC: 0.557
Sensitivity
Specificity

(b)

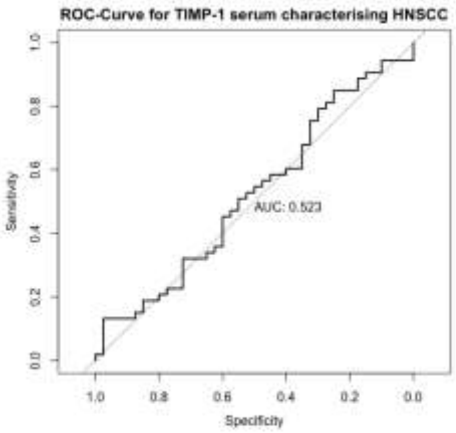
ROC-Curve for TIMP-1 serum characterising HNSCC
AUC: 0.523
Sensitivity
Specificity

(b)

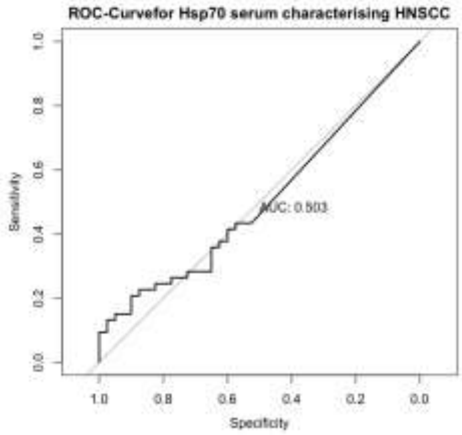
ROC-Curvefor Hsp70 serum characterising HNSCC
AUC: 0.503
Sensitivity
Specificity

(d)

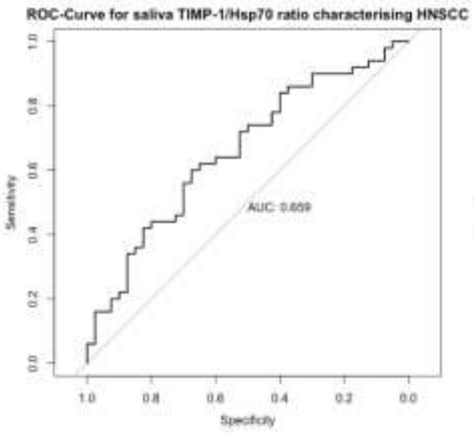
ROC-Curve for saliva TIMP-1/Hsp70 ratio characterising HNSCC
AUC: 0.659
Sensitivity
Specificity

(e)

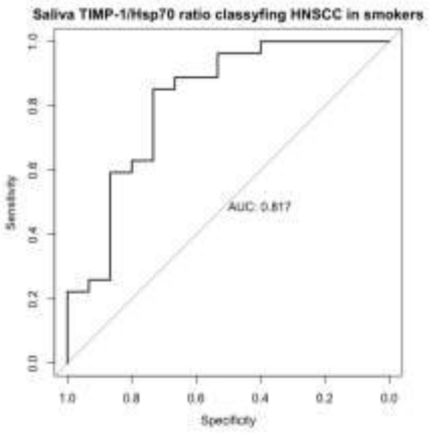
Saliva TIMP-1/Hsp70 ratio classyfing HNSCC in smokers
AUC: 0.817
Sensitivity
Specificity

(f)

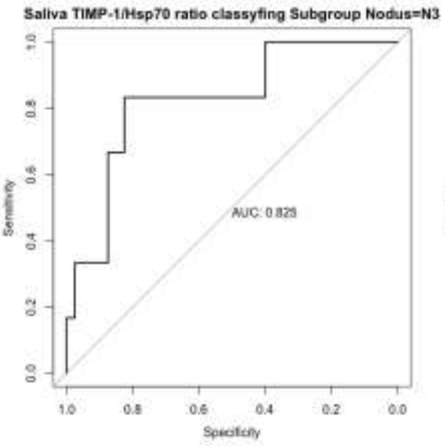
Saliva TIMP-1/Hsp70 ratio classyfing Subgroup Nodus=N3
Sensitivity
Specificity
AUC: 0.825

(g)

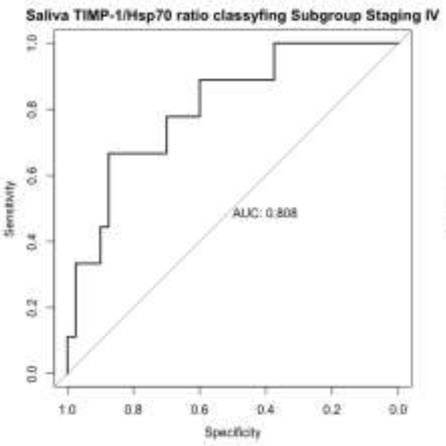
Saliva TIMP-1/Hsp70 ratio classyfing Subgroup Staging IV
Sensitivity
Specificity
AUC: 0.808

(h)

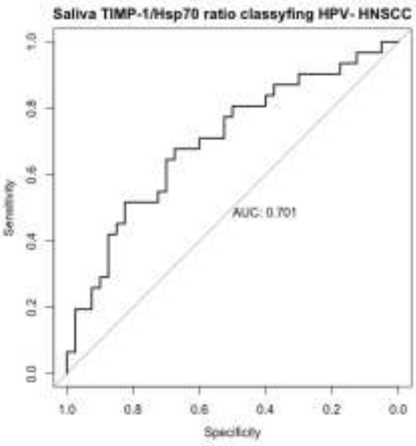
Saliva TIMP-1/Hsp70 ratio classyfing HPV- HNSCC
Sensitivity
Specificity
AUC: 0.701

(i)

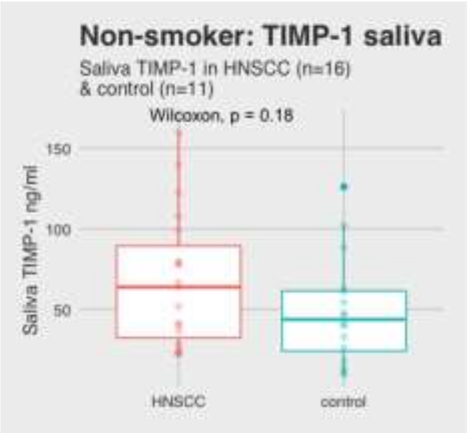
Non-smoker: TIMP-1 saliva
Saliva TIMP-1 in HNSCC (n=16)
& control (n=11)
Wilcoxon, p = 0.18
Saliva TIMP-1 ng/ml
HNSCC
control

(j)

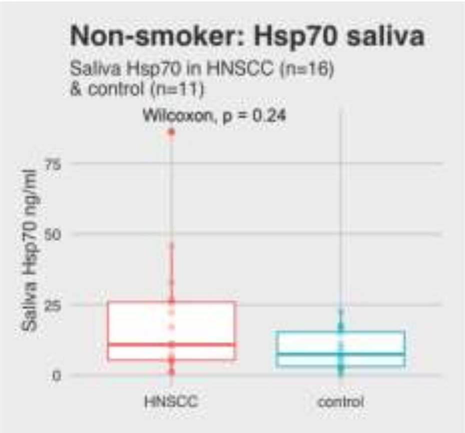
Non-smoker: Hsp70 saliva
Saliva Hsp70 in HNSCC (n=16)
& control (n=11)
Wilcoxon, p = 0.24
Saliva Hsp70 ng/ml
HNSCC
control

(k)

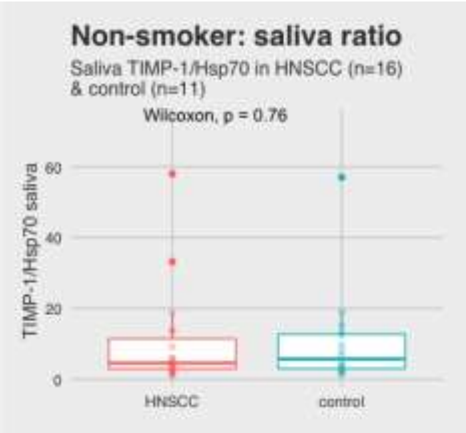
Non-smoker: saliva ratio
Saliva TIMP-1/Hsp70 in HNSCC (n=16)
& control (n=11)
Wilcoxon, p = 0.76
TIMP-1/Hsp70 saliva
HNSCC
control

(l)

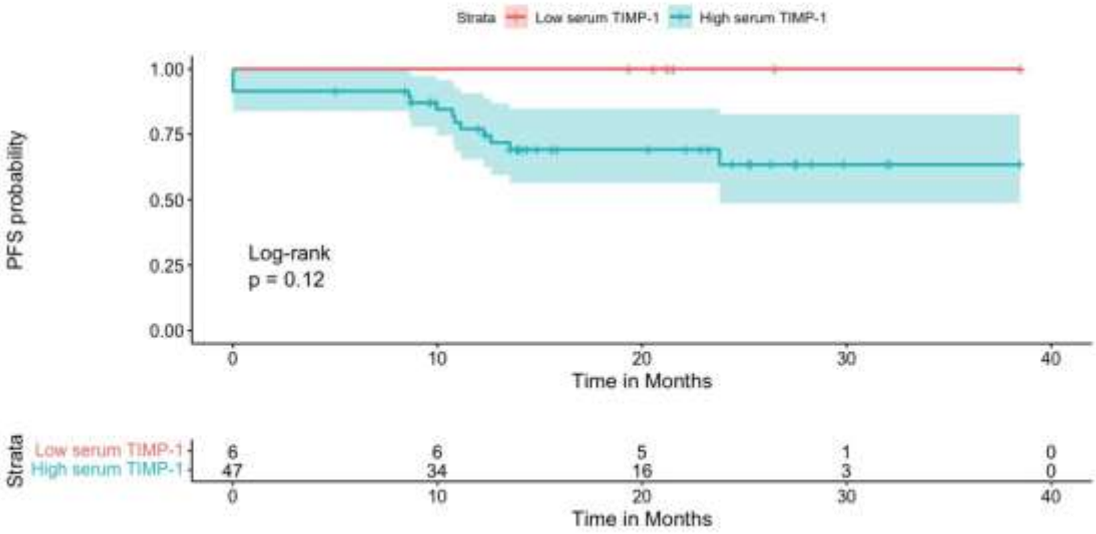
Strata
Low serum TIMP-1
High serum TIMP-1
PFS probability
Log-rank
p = 0.12
Time in Months
Strata
Low serum TIMP-1 6 6 5 1 0
High serum TIMP-1 47 34 16 3 0
Time in Months

(m)

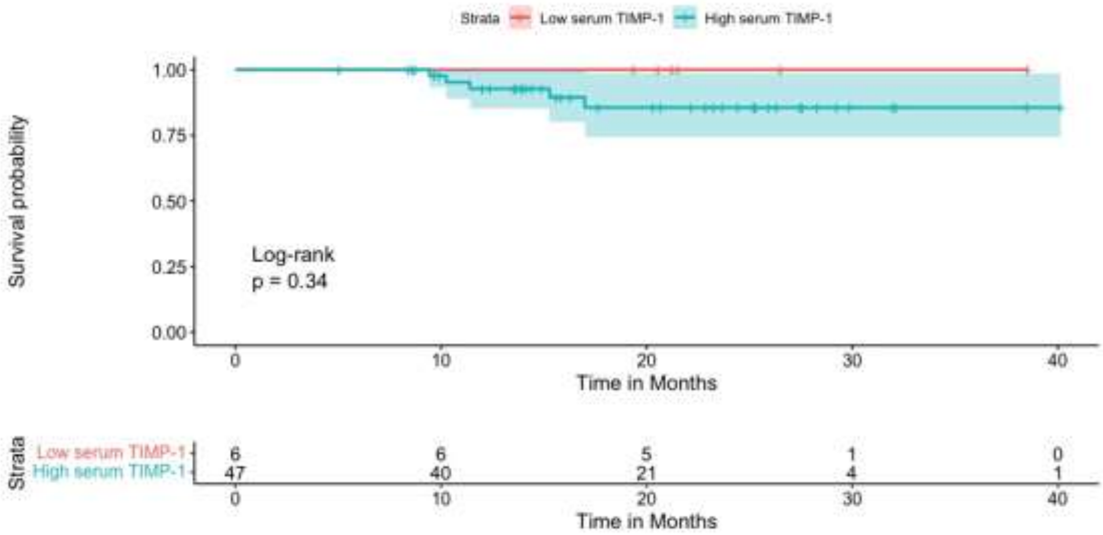
Strata Low serum TIMP-1 High serum TIMP-1
Survival probability
1.00
0.75
0.50
0.25
0.00
Log-rank
p = 0.34
0
10
20
30
40
Time in Months
Strata
Low serum TIMP-1 6 6 5 1 0
High serum TIMP-1 47 40 21 4 1
Time in Months

(n)

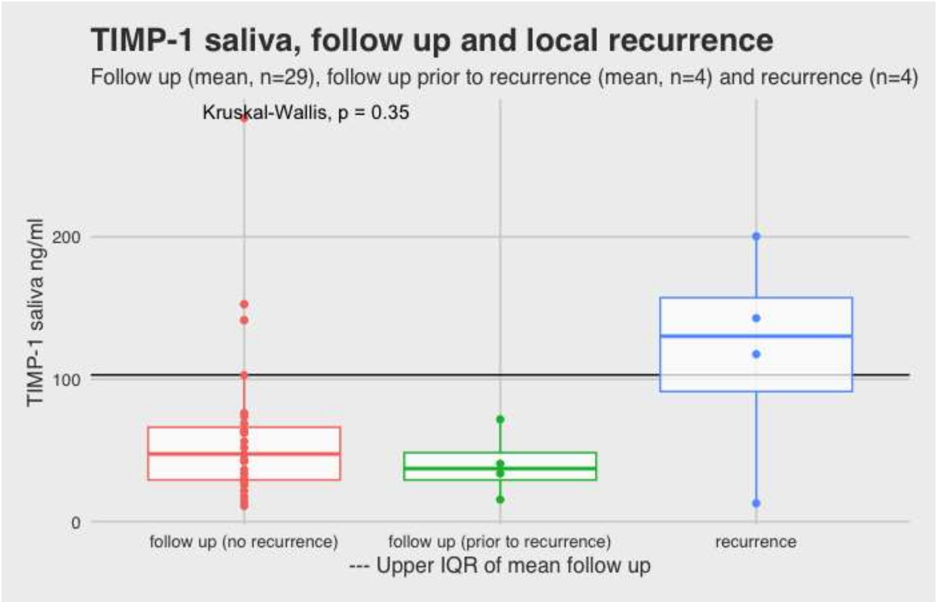
TIMP-1 saliva, follow up and local recurrence
Follow up (mean, n=29), follow up prior to recurrence (mean, n=4) and recurrence (n=4)
Kruskal-Wallis, p = 0.35
TIMP-1 saliva ng/ml
200
100
0
follow up (no recurrence)
follow up (prior to recurrence)
recurrence
--- Upper IQR of mean follow up

(o)

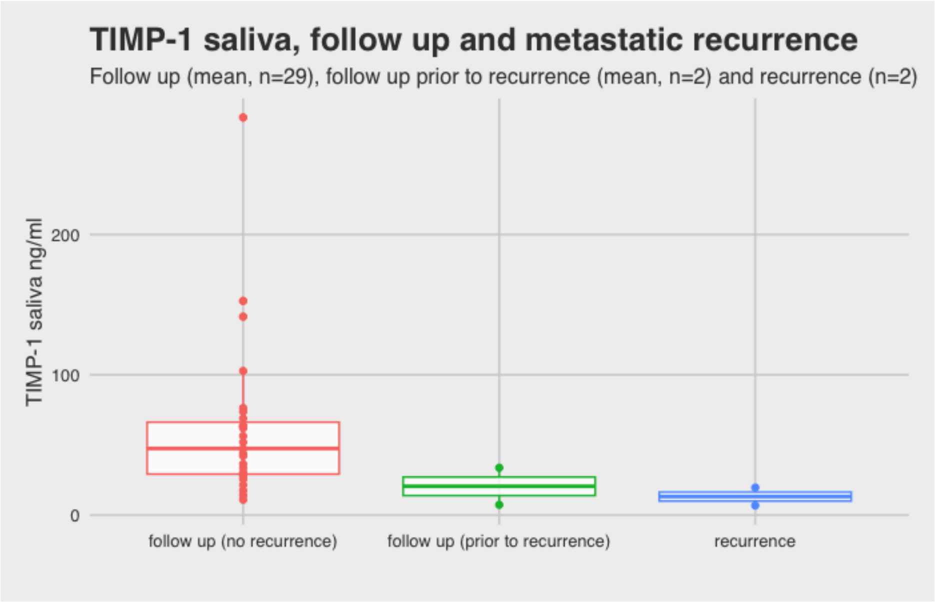
TIMP-1 saliva, follow up and metastatic recurrence
Follow up (mean, n=29), follow up prior to recurrence (mean, n=2) and recurrence (n=2)
TIMP-1 saliva ng/ml
200
100
0
follow up (no recurrence)
follow up (prior to recurrence)
recurrence

(p)

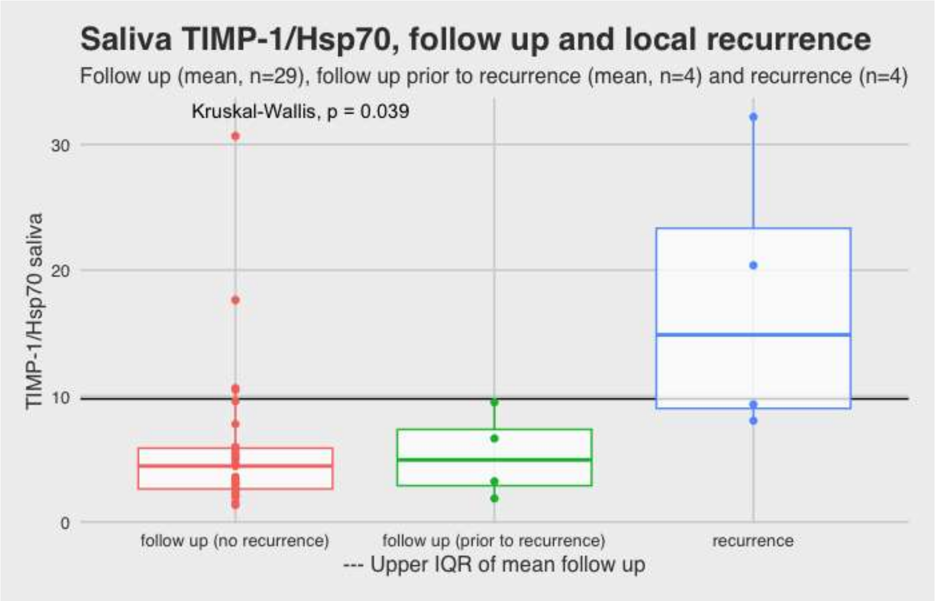
Saliva TIMP-1/Hsp70, follow up and local recurrence
Follow up (mean, n=29), follow up prior to recurrence (mean, n=4) and recurrence (n=4)
Kruskal-Wallis, p = 0.039
TIMP-1/Hsp70 saliva
30
20
10
0
follow up (no recurrence)
follow up (prior to recurrence)
recurrence
--- Upper IQR of mean follow up

(q)

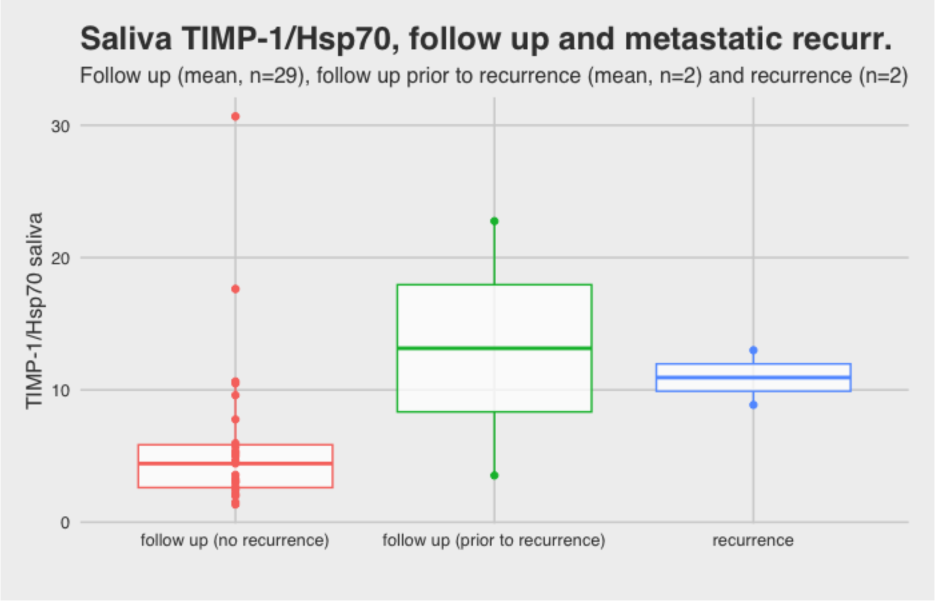

(r)

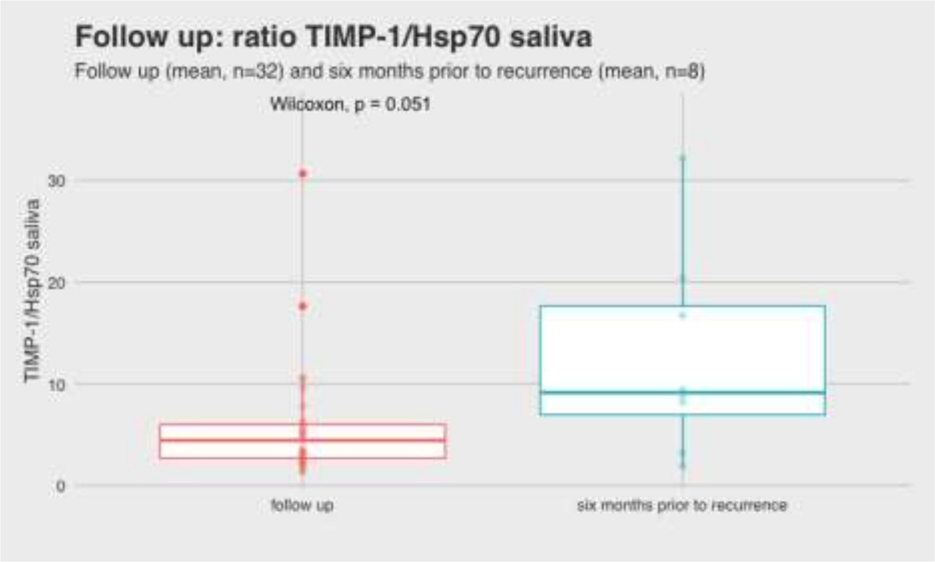

(s)

Supplementary Figure S1. (a) Receiver operator characteristic curve for saliva TIMP-1 classifying HNSCC patients (*n*=53) and control subjects (*n*=40), (b) Receiver operator characteristic curve for saliva Hsp70 classifying HNSCC patients (*n*=53) and control subjects (*n*=40), (c) Receiver operator characteristic curve for serum TIMP-1 classifying HNSCC patients (*n*=53) and control subjects (*n*=40), (d) Receiver operator characteristic curve for serum Hsp70 classifying HNSCC patients (*n*=53) and control subjects (*n*=40), (e) Receiver operator characteristic curve for TIMP-1/Hsp70 saliva ratio classifying HNSCC patients (*n*=53) and control subjects (*n*=40), (f) Receiver operator characteristic curve for TIMP-1/Hsp70 saliva ratio classifying HNSCC patients in smoker subgroup with smokers with HNSCC (*n*=28) & smokers control (*n*=15) (g) Receiver operator characteristic curve for TIMP-1/Hsp70 saliva ratio classifying HNSCC patients in a subgroup with Nodus state N3 (*n*=6) & control (*n*=40), (h) Receiver operator characteristic curve for TIMP-1/Hsp70 saliva ratio classifying HNSCC patients in a subgroup with UICC Stage IV (*n*=6) & control (*n*=40), (i)

Receiver operator characteristic curve for TIMP-1/Hsp70 saliva ratio classifying HNSCC patients in a subgroup with HPV negative (*n*=34) & control (*n*=40), (j) Saliva ratio TIMP-1 in non-smoking subjects, comparing HNSCC and the combined control group, (k) Saliva ratio Hsp70 in non-smoking subjects, comparing HNSCC and the combined control group, (l) Saliva ratio TIMP-1/Hsp70 in non-smoking subjects, comparing HNSCC and the combined control group, (m) PFS probability for TIMP-1 concentration above (high) and below (low) cut off value 196ng/ml, (n) OS probability for TIMP-1 concentration above (high) and below (low) cut off value 196ng/ml, (o) Saliva TIMP-1 concentration mean during follow up, follow up before local recurrence and concentration at diagnosis of local recurrence, (p) Saliva TIMP-1/Hsp70 ratio mean during follow up, follow up before local recurrence and at diagnosis of local recurrence, (q) Saliva TIMP-1/Hsp70 ratio mean during follow up, follow up before metastatic recurrence and concentration at diagnosis of metastatic recurrence, (r) Saliva TIMP-1/Hsp70 ratio mean in follow up, follow up before metastatic recurrence and concentration at diagnosis of metastatic recurrence, (s) TIMP-1/Hsp70 saliva ratio mean during follow up and mean six months prior to recurrence
